# Morphological and Genetic Diversity of the Wood-Boring *Xylophaga* (Mollusca, Bivalvia): New Species and Records from Deep-Sea Iberian Canyons

**DOI:** 10.1371/journal.pone.0102887

**Published:** 2014-07-25

**Authors:** Chiara Romano, Janet Ruth Voight, Rocío Pérez-Portela, Daniel Martin

**Affiliations:** 1 Centre d'Estudis Avancats de Blanes (CEAB - CSIC), Blanes (Girona), Catalunya, Spain; 2 Integrative Research Center, The Field Museum, Chicago, Illinois, United States of America; Laboratoire Arago, France

## Abstract

Deep-sea bivalves of the Xylophagaidae, a poorly known group, are obligate wood-borers. Deployment of wood in three submarine canyons off the Iberian coast, the Blanes and La Fonera Canyons (Mediterranean Sea) and the Avilés Canyon (Cantabric Sea, Bay of Biscay), lead to the discovery of four xylophagaid species in our samples. *Xylophaga dorsalis* (the dominant species), *X. atlantica, X.* cf. *anselli* and the new species *X. brava*, were identified on the basis of morphological data, and supported by a phylogenetic reconstruction based on the nuclear genes 18S rDNA and 28S rDNA and including several genus of Xylophagaidae. Genetic divergence between species of *Xylophaga* varied between genes, ranging from 0.5 to 4.0% for the 18SrDNA and from 4.1 to 16.6% for the 28SrDNA. *Xylophaga brava* sp. nov. appeared to be restricted to the Mediterranean and morphologically resembled the closely related *X.* cf. *anselli* from the Cantabrian Sea. However, they clearly diverged in two well-supported clades. Low levels of intraspecific variability and higher interspecific divergence between species also supported the existence of these two different species. Morphologically they differ in the number of cirri at the siphon openings, in the shape of the posterior shell and in the size of prodissoconch II. The new species is characterized by having weak, poorly mineralized mesoplax and siphons united throughout, covered by a periostracal, non-calcified tube; distinct proximal and distal siphons, the former translucent and soft, the latter muscular, with concentric rings. *Xylophaga atlantica,* previously known only from the western Atlantic, is reported for the first time in the Mediterranean Sea. Whether its presence in the Mediterranean indicates its natural distribution or reflects its recent introduction is unknown. Although xylophagaids have been previously reported to recruit heavily to wood deposited on the seabed, these four species colonized wood suspended 30 m above the seafloor.

## Introduction

Bivalves in the genus *Xylophaga* Turton, 1922 are obligate wood-borers in the deep sea. Their specialized shell carries denticles on their anterior beak to scrape wood, boring a hole in which they live, and their large, U-shaped diverticulum ofthe stomach accumulates wood shavings [1]. Symbiotic bacteria had been observed in their gills [2] but not yet cultivated and characterized. With limited sampling, the level of species diversity of xylophagaids is surprisingly high [3]. However, most species have been collected only once [4], [5] and very little is known of their distribution, dispersal potential and other biological features.

The Mediterranean Sea is among the world's best known marine areas [6], however its deeper water fauna is largely unexplored. No species of *Xylophaga* have been formally described from Mediterranean specimens, although their presence had previously been reported without comment (e.g.[7], [8]). In total, only five xylophagaid species are known from the entire eastern Atlantic Ocean, including the Mediterranean Sea [4].

Submarine canyons are widely distributed along the ocean margin and more closely spaced in the Mediterranean than in other areas [9].They contribute substantially to channelling matter from the shore to deep basins [10], [11] and often sustain high biodiversity and biomass of many benthic faunal groups [12]–[15]. However, few studies on macrobenthic fauna have focused in Mediterranean submarine canyons [15], [16]. The organic material concentrated on the submarine canyon floors may include sunken wood and other vegetation. Consequently canyons may offer ideal habitats for wood-dependent xylophagous bivalves, as suggested by the higher abundance of *Xylophaga* specimens inside a NW Mediterranean canyon than on the adjacent open slope [17]. Nevertheless, the taxonomic identification of the wood-boring organisms has not been pursued, despite recent studies on sunken wood in the Mediterranean [17]–[21].

**Figure 1:**
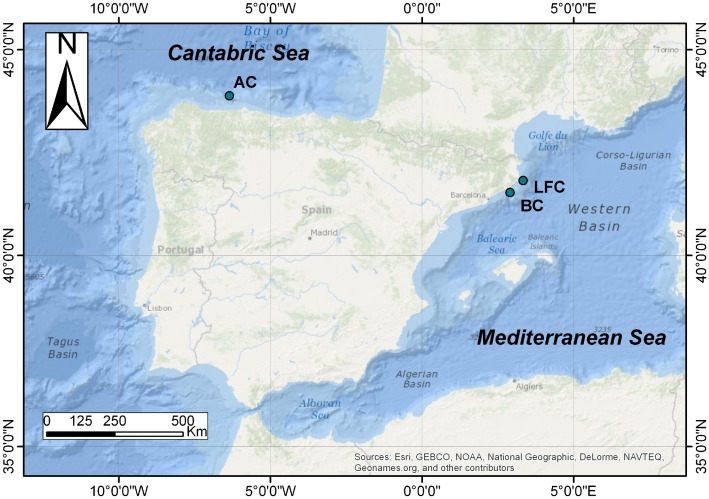
Map showing the Blanes Canyon (BC), La Fonera Canyon (LFC) and Avilés Canyon (AC) and location of deployments along the Iberian Peninsula. Map was modified from Romano et al. 2013.

Given the lack of available information about members of *Xylophaga* along the Iberian Peninsula, and in general in the Mediterranean Sea, the present study intends to: i) increase our knowledge of the diversity and distribution of the xylophagaids in three submarine canyons of the Iberian Peninsula (Blanes and La Fonera at the NW Mediterranean Sea and Avilés at the Cantabrian Sea in the Atlantic Ocean, Fig. 1), ii) formally describe a new species of *Xylophaga* from the Mediterranean deep sea, and iii) improve taxonomic resolution for *Xylophaga* species combining, for the first time for this genus, morphological with molecular methods. The application of molecular tools complements morphological analyses, and clarifies species that are difficult to distinguish based only on morphology [22]. Molecular analyses may be especially useful for wood-boring bivalves because little information about within-species phenotypic plasticity is available, and specimens of this group are often extracted incomplete from the wood, making species identification from morphological features difficult.

## Material and Methods

### Sampling areas

Blanes (BC) and La Fonera (LFC) Canyons are shelf-incised, long canyons at the Catalan margin of the Northwest Mediterranean Sea, along the “Costa Brava” shoreline (Fig.1). Their heads are separated by almost 38 km of relatively shallow shelf (Fig. 1). BC is north–south oriented; it extends in total 184 km to the Channel of Valencia at approximately 2600 m depth [23]. LFC, oriented almost NW–SE, extends 110 km long to a maximum depth of approximately 2550 m [24], [25]. Heads of both canyons are about 4 km from the coast, near the mouths of the Tordera and Ter Rivers, respectively. BC and LFC are key areas for the recruitment and maintenance of economically relevant biological resources, such as the rose shrimp *Aristeus antennatus* (Risso, 1816), [26] and are hotspots for biomass concentration and elevated biodiversity [17], [27]–[29].

Avilés Canyon (AC) is one of the world's deepest canyons. Located on the Bay of Biscay (commonly named Cantabric Sea) platform (NE Atlantic, Spain, Fig. 1), it is about 2,400 km from BC. Its head (140 m deep) is 13 km from the coast and its maximum depth is 4750 m [30]. AC is an essential habitat for a highly diverse benthic community [30] including reproductive stages of commercially important species such as the sardine, *Sardina pilchardus* (Walbaum, 1792), [31], and for many different marine predators such as seabirds, cetaceans and the giant squid, *Architeuthis dux* Steenstrup, 1857 [32].

### Experimental deployments

Wood deployments were submerged in the three canyons described above (Fig. 1). Each deployment consisted of three replicates cubes (8 cm on a side) or pieces (13×13×3 cm) of each of the most common wood in adjacent watersheds, pine and oak. Each wood piece was enclosed by plastic net bag with a 0.5 cm mesh. This allowed juveniles to settle but minimized losses during recovery. Each deployment was fixed to mooring lines (see[33] for full description), 20–40 m above the anchor weights; floats kept them above the substrate. Except for the shallowest, deployments were dropped from the surface and recovered when an acoustic release freed the mooring lines, deployment, and floats from the anchor weight. The shallowest deployment (LFC 130 m deep) rested on the sediment and was recovered after 10 months by an ROV (Remotely Operated Vehicle).

Inside BC, a deployment was made at intervals of 300 m depth from 900 to 1500 m; a similar series of deployments was made on the canyon's adjacent open slope (BC-OS) from 1200 to 1800 m depth, 30 km to the west. In LFC, deployments were made at 130 m and 1100 m depth in the main axis of the canyon. In AC, a deployment was made at 2000 and 4700 m depth inside the canyon and one was made at 1200 m depth on its western slope (AC-WS). In BC and BC-OS at 1200 m depth we collected two deployments (a, b), after 3 and 9 months of immersion respectively. In the other sites, deployments remained in place from 7 to 12 months (Table 1).

**Table 1:** Location and deployment time of the wood traps in the Blanes Canyon (BC), in the adjacent open slope (BC-OS), in the La Fonera Canyon (LFC), in the Avilés Canyon (AC) and its adjacent western slope (AC-WS).

Trap	Seafloordepth (m)	Latitude	Longitude	Drop date	Sampling date	Duration(months)	*Xylophaga* species
BC 900	894	41° 34' 12.72'' N	2° 50' 19.14'' E	November 2008	November 2009	12	Xat - Xbr - Xdo
BC 1200a	1190	41° 31' 15'' N	2° 50' 50'' E	November 2008	February 2009	3	Xdo
BC 1200b	1195	41° 31' 15.06'' N	2° 50' 49.26'' E	February 2009	November 2009	9	Xat - Xbr - Xdo
BC 1500	1468	41° 27' 28.80'' N	2° 52' 58'' E	November 2008	November 2009	12	Xat - Xbr - Xdo
BC-OS 1200a	1180	41° 13' 9.2'' N	2° 48' 55'' E	November 2008	February 2009	3	Xdo
BC-OS 1200b	1184	41° 13' 8,99'' N	2° 48' 54.6'' E	February 2009	November 2009	9	Xat - Xdo
BC-OS 1500	1497	41° 09' 0.59'' N	2° 53' 48'' E	November 2008	November 2009	12	Xat - Xbr - Xdo
BC-OS 1800	1806	41° 04' 52.19'' N	2° 58' 9'' E	November 2008	November 2009	12	Xat - Xbr - Xdo
LFC 130	135	41° 53' 27.25" N	3° 14' 45.2" E	June 2010	April 2011	10	Xdo
LFC 1100	1150	41° 51' 51" N	3° 20' 10.2" E	March 2012	October 2012	7	Xat - Xbr - Xdo
AC 2000	2100	43° 54' 37.3" N	6° 21' 0.1" W	March 2012	September 2012	7	Xat -Xdo - Xans
AC 4700	4726	44° 07' 47.9" N	6° 14' 22.32" W	March 2012	September 2012	7	-
AC-WS 1200	1257	43° 58' 06.9" N	6° 30' 50.9" W	March 2012	September 2012	7	Xat - Xdo- Xans

*Xylophaga* species in each deployment are also reported. Xat  =  *X. atlantica*, Xbr =  *X. brava* n.sp, Xdo =  *X.dorsalis, Xans =  X. cf. anselli*.

No specific permissions were required for deploying submerged moorings in Blanes, La Foneara and Avilés Canyons, as they are not protected areas, moreover this study did not involve endangered or protected species.

### Sample treatment and morphological observations

**Figure 2:**
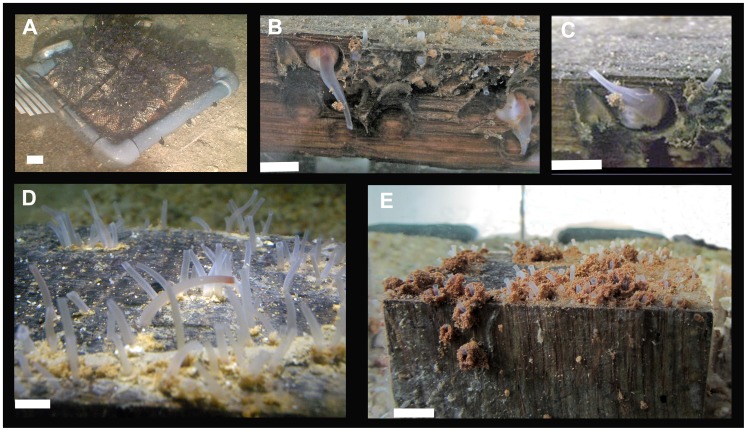
Experimental wood samples: A) on the seafloor at LFC at 130 m depth before recovery (artificially lit by ROV); B), C) Individuals of *X. dorsalis* in the aquarium in wood that was recovered after 10 months from 130 m deep in LFC; D) *Xylophaga* spp. the day after recovery from La Fonera Canyon (1100 m deep) in wood after 6 months immersion; E) oak from La Fonera Canyon 1100 m one week after recovery. Note the development of fecal chimneys around the siphons.

Immediately after recovery, two blocks of oak and of pine from each deployment were fixed in 4% buffered formaldehyde-seawater solution; the others were covered in absolute ethanol until laboratory analysis. Two blocks from two Mediterranean deployments (LFC 130 and 1100 m depth) were immediately immersed in seawater in refrigerated tanks (13°C) to observe the behaviour of *Xylophaga* spp. (Fig. 2). In the laboratory, the formalin-preserved blocks were washed with distilled water. All wood blocks were photographed, sectioned by hand, and then carefully dissected with the aid of a magnifier (2 X) or a dissecting microscope to count all xylophagaids. The specimens obtained were either stored in absolute ethanol or, if fixed in formalin, in 70% ethanol for further morphological observations. However, we do not expect preservation artifact to impact our results because different recoveries were treated in the same manner. Measurements were made to the nearest 0.1 mm using electronic calipers. Specimens were grouped by morphotype into 5 groups (A to E following Romano et. al. [17]), plus morphotype F that corresponds to some xylophagaid specimens collected in AC.

Light microscope micrographs were made with a CT5 digital camera (Jenoptics, Jena) attached to a SMZ1000 Nikon stereomicroscope using the software Capture Pro 2.8.8.1 © by Jenoptics 2011. Illustrations were made with a Wacom digitizing tablet, using C5 Adobe Illustrator (15.0.2) and Photoshop (version 12.0.4) © by Adobe Systems Inc. 2010. Scanning Electron Microscope (SEM) micrographs were made with a Hitachi TM 3000 Tabletop electron microscope, at the SEM service of the Centre d'Estudis Avancats de Blanes (CEAB), based on ethanol-preserved specimens dried at 50 to 60°C.

Specimens of *Xylophaga dorsalis* (Turton, 1819) from Natural History Museum, London (BMNH), the Plymouth City Museum and Art Gallery (PCMAG), the Musée Océanographique de Monaco (MOM), the Landeshauptstadt Düsseldorf (LHD), the Marine Biological Association (MBA) were used for comparison. Specimens of *X. anselli* from The Field Museum of Chicago (FMNH) were also analysed. Type specimens and additional material from the present study were deposited at FMNH and at the Museo Nacional de Ciencias Naturales de Madrid (MNCN).

### DNA extraction, amplification, and sequencing

Xylophagaid specimens of four different morphotypes were randomly selected for genetic analyses (Table 2) from ethanol-preserved samples. Only one individual of morphotype D was available for genetic analyses, and no suitably ethanol- preserved material of morphotype E was available. Analyses were also conducted on specimens of *X. dorsalis* (morphotype A): three from PCMAG (ID 2000.1.-2921.2;.3161.1; 3168.2), one specimen from the LHD, and two from MBA collected near the species type locality.

**Table 2:** Geographical location and GEnBank accession numbers of taxa and sequences used for this study.

Species	ID	Morphotype	Location, Depth (m)	GenBank accession 18S	GenBank accession 28S
*Xylophaga dorsalis*	4	A	BC, 1200	KJ946323	KJ946340
*Xylophaga dorsalis*	29	A	LFC, 130	KJ946324	KJ946341
*Xylophaga dorsalis*	30	A	LFC, 130		KJ946349
*Xylophaga dorsalis*	31	A	AC-WS, 1200	KJ946321	KJ946346
*Xylophaga dorsalis*	32	A	AC, 1200	KJ946322	
*Xylophaga brava n. sp.*	7	B	BC, 1200	KJ946325	KJ946335
*Xylophaga brava n. sp.*	8	B	BC, 1200	KJ946326	KJ946336
*Xylophaga brava n. sp.*	9	B	BC, 1200	KJ946327	KJ946337
*Xylophaga brava n. sp.*	18	B	BC, 1200	KJ946328	
*Xylophaga brava n. sp.*	36	B	LFC, 1100	KJ946329	
*Xylophaga brava n. sp.*	37	B	LFC, 1100	KJ946330	
*Xylophaga atlantica*	19	C	BC, 1200	KJ946316	KJ946339
*Xylophaga atlantica*	26	C	BC, 1200	KJ946317	KJ946342
*Xylophaga atlantica*	38	C	LFC, 1100	KJ946318	KJ946338
*Xylophaga atlantica*	39	C	LFC, 1100	KJ946319	KJ946344
*Xylophaga atlantica*	41	C	LFC, 1100	KJ946315	KJ946345
*Xylophaga atlantica*	40	D	LFC, 1100	KJ946320	KJ946343
*Xylophaga* cf. *anselli*	86	F	AC 2000	KJ946332	KJ946350
*Xylophaga* cf. *anselli*	89	F	AC, 2000	KJ946331	KJ946347
*Xylophaga* cf. *anselli*	95	F	AC, 2000	KJ946333	
*Xylophaga* cf. *anselli*	98	F	AC, 2000	KJ946334	KJ946348
*Xylophaga atlantica*			12 miles east of Southwest Harbor, ME, USA 100	AY070123	AY070132
*Xylophaga sp.*			SE of Port Dundorf, South Africa (29°02.2'S, 32°19.6'E) 800 m	JF899226	JF899198
*Xylophaga washingtona*			Dredged wood, Friday Harbor, WA, USA	JF899227	JF899199
*Xylopholas sp.*			Gulf of Mexico (27°44.75'N, 91°13.31'W) 540 m	JF899228	JF899200
*Xyloredo sp.*			Gulf of Mexico (27°44.75'N, 91°13.31'W) 540 m	JF899229	JF899201
*Barnea candida*			Whitstable, UK	AM774541	AM779715
*Barnea truncata*			Wachapreague, VA USA	JF899206	JF899178
*Mya arenaria*			Pavillion Beach, Ipswich, MA, USA	AF120560	FM999792

Sequences of *Xylophaga* spp. determined in this investigation are indicated with an individual code (ID). Samples from this study are from NW Mediterranean canyons (Blanes Canyon, BC; La Fonera Canyon, LFC) except 31-32, which are from Cantabric Sea canyon (Avilés Canyon AC) (NE Atlantic Ocean).

DNA was extracted from muscle (siphon or foot) using the DNeasy Mini Kit (Qiagen, www.qiagen.com). Fragments of the nuclear 28S rDNA and 18S rDNA genes (henceforth 28S and 18S, respectively) were amplified and sequenced by polymerase chain reaction with the primers used by Distel [41]: 18S-EukF, 18S-EukR, 28S-NLF184-21 and 28S-1600R as listed in Table 3. PCR amplification reactions were performed in a 50 µl of total reaction volume with 1 µl of each primer (10 µM), 1.5 µl dNTPs (10 µM), 5 µl buffer, 0.75 µl MgCl_2_, 1 µl Bovine Serum Albumin, 0.7 Taq polymersase (Genotek), 37.05-38.05 µl milliQ water, and 1-2 µlDNA template. PCR program consisted in a single step at 94°C for 5 min followed by 35 cycles (denaturation at 94°C for 1 min, annealing at 50–53°C for 1 min and extension at 72°C for 1 min) and a final extension at 72°C for 5 min on a thermal cycler (BioRad MyCycler). PCR products were purified and sequenced in both directions with the forward and reverse primers, with an ABI Bigdye v3.1 Cycle Sequencing Kit on a 3730XL DNA Analyzer at Macrogen (http://www.macrogen.com/eng/). All sequences generated are deposited in Genbank (Table 2).

**Table 3:** Sequences of the primers used for amplification and sequencing in this study.

Target-Primer Name	Primer Sequence (5′ → 3′)	Sense	References
18S-EukF	WAYCTGGTTGATCCTGCCAGT	Forward	Medlin et al., 1988
18S-EukR	TGATCCTTCYGCAGGTTCACCTAC	Reverse	Medlin et al., 1988
28S-NLF184-21	ACCCGCTGAAYTTAAGCATAT	Forward	www.psb.urgent.be/rRNA
28S-1600R	AGCGCCATCCATTTTCAGG	Reverse	Distel et al., 2011

### Molecular analyses

Consensus sequences for each individual and gene were obtained from forward and reverse sequences, following basic editing using Bioedit Sequence Alignment Editor v. 7.0.9.1 [34]. All sequences (28S and 18S) were submitted to BLAST searches in the GenBank database to ensure that symbiont bacteria and other potential contaminants had not been erroneously co-amplified. To the dataset of sequences of *Xylophaga* spp. that we generated, we added sequences of the 28S and 18S from Genbank (Table 2) for other *Xylophaga* species, for the related genera *Xyloredo* and *Xylopholas*, and *Barnea*, *Bankia*, and *Mya* species as outgroups. Sequences were aligned using MUSCLE as implemented in SEAVIEW 4.4.2 [35] and confirmed by eye. Final sequence lengths after trimming and alignment were 1107 bp and 1403 bp for 28S and 18S, respectively.

For analysis, the individual data sets of 28S and 18S were separately run under the BI (Bayesian Inference) method. Both markers were then concatenated and BI and Maximum Likelihood (ML) methods were applied for phylogenetic reconstruction. The best-fit models of nucleotide substitution were selected by statistical comparison of 812 different models of evolution with the program jmodeltest2 [36] under the Akaike Information Criterion (AIC). For BI analyses we used MrBayes 3.1.2 [37].Values of the evolution models selected were input in the software, and two million generations were executed with a sample frequency of 100 (20,000 final trees). After verifying that stationarity had been reached, both in terms of likelihood scores and parameter estimation, the first 5,000 trees were discarded, and independent majority-rule consensus trees were generated from the remaining (15,000) trees. For ML the models selected were input into RAxML [38] and nodal supports were assessed by 500 bootstrap replicates. The appropriate partitions were defined for the concatenated analyses, and the different substitution models applied.

For species delimitation we used a Poisson Tree Processes model (PTP) on the rooted phylogenetic trees of the 28S and 18S, and as implemented in the PTP web server. Genetic divergences between and within species were calculated as p-distances with the software MEGA v 5.1 [39].

### Nomenclatural Acts

The electronic edition of this article conforms to the requirements of the amended International Code of Zoological Nomenclature, and hence the new names contained herein are available under that Code from the electronic edition of this article. This published work and the nomenclatural acts it contains have been registered in ZooBank, the online registration system for the ICZN. The ZooBank LSIDs (Life Science Identifiers) can be resolved and the associated information viewed through any standard web browser by appending the LSID to the prefix "http://zoobank.org/". The LSID for this publication is: urn:lsid:zoobank.org:pub:390E5C26-92C2-4EA4-A39C-2D244829AFAF. The electronic edition of this work was published in a journal with an ISSN, and has been archived and is available from the following digital repositories: PubMed Central, LOCKSS and Digital.CSIC.

## Results

All the deployed wood pieces were colonized by *Xylophaga* specimens (Fig. 2), except for those collected at the deepest AC station (AC 4700) that appeared to be intact and were not colonized by any macroinvertebrate.

The six morphotypes (A to F) identified are described in the morphological section below. The shallowest deployment (LFC 130) and the three-month samples (BC and BC-OS 1200) were colonized only by morphotype A; more than one species co-occurred in all other deployments (Table 1). A total of 3502 specimens of *Xylophaga* were examined. Morphotype A was dominant (83%), followed by morphotype B (14%); the other morphotypes were recovered at dramatically lower frequencies (each one at <1%).

Within a few days of being placed in the aquarium, faecal chimneys surrounding and covering the siphons of the living *Xylophaga* spp. were seen outside the wood (Fig. 2E).

### Molecular analyses

Twenty-two specimens from five morphotypes from different canyons were successfully sequenced (Table 2). Attempts to obtain sequences from museum specimens of *Xylophaga dorsalis* from LHD, PCMAG and MBA were unsuccessful.

In total, 29 (for 28S) and 33 (for 18S) sequences (Tab. 2) were analysed to reconstruct phylogenetic trees for both genes (data not shown). Nevertheless, the final tree from the concatenated dataset included 2510 bp from both genes and 26 specimens. In the Modeltest procedure, the AIC indicated that the GRT + G + I was the best-fit model for 28S sequences, and the TrN + G + I was the best-fit model among those evaluated for 18S sequences.

**Figure 3:**
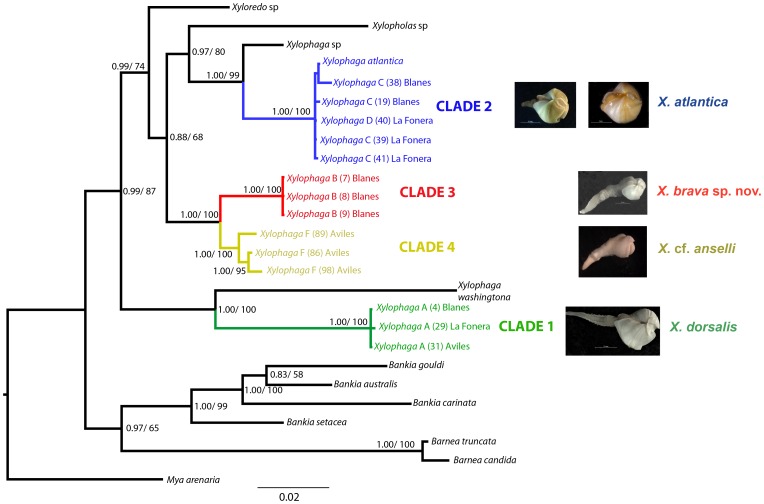
Bayesian Inference (BI) tree, including Maximum Likelihood (ML) bootstrap supports on the nodes, generated from sequences of the18SrDNA and 28S rDNA genes. Values of support below 0.5 and 50% are not shown. The scale bar represents the number of nucleotide substitutions per site. Letters (A, B, C and D) after the genus name correspond to the “morphotype” of *Xylophaga*, and numbers in brackets to the individual codes (Table 2).

The phylogenetic trees obtained by BI and ML of the 28S and 18S concatenated sequences (Fig. 3) showed basically the same topology, and the monophyly of the Xylophagaidae, as major and well-supported clade (posterior probabilities of 0.99 and 87% of bootstrap support).

Moreover the phylogenetic tree recovered our specimens of *Xylophaga* as forming four well-supported clades (posterior probabilities of 1.00 and 100% of bootstraps), namely Clade 1, Clade 2, and Clade 3 and Clade 4. However, species of *Xylophaga* appeared separated in two main groups: one clade included *X. washingtona* and Clade 1, and the second that grouped four species of *Xylophaga*. This second group also separated two subclades; *Xylophaga* sp. and Clade 2 as sister species together with a sequences of *Xylopholas* sp. from Genbank, and Clade 3 and Clade 4 as closely related species. The species *Xyloredo* sp. obtained from Genbank also appeared included within this second group but separated from the *Xylophaga* species (Fig. 3).

Clade 1 corresponds to morphotype A from all three canyons; specimens from the two geographically close canyons and the third distant canyon were functionally inseparable based on the sequenced genes (Fig. 3). These are identified as *X. dorsalis* in the systematic account. Clade 2 corresponds to morphotype C from BC and LFC and to a small specimen of morphotype D from LFC, which were genetically indistinguishable from each other and from the sequence of *X. atlantica* Richards, 1942 deposited in GenBank [40], [41]. Clade 3 corresponds to morphotype B from BC and LFC, which is formally described as a new species, *X. brava,* in the systematic account of this paper. Finally, Clade 4 appears to be a different and well-supported cluster, more closely related to Clade 3 than other clades, and including morphotype F from AC, which is identified as *X*. cf. *anselli* in the systematic account.

Genetic divergence among the four clades was congruent with the observed morphological differences. Levels of divergence were higher in the 28S sequences than in 18S (Table 4). Sequences of *X. washingtona,* obtained from Genbank, showed the greatest genetic distances with the other species for both genes.

**Table 4:** Pairwise genetic divergence for *Xylophaga* species.

	*X. washingtona* n = 1	*X. dorsalis* n = 5	*X. brava* sp. nov. n = 6	*X. atlantica* n = 6	*X.* cf. *anselli* n = 4	Within species (18SrDNA)
*X. washingtona* n = 1	-	0.036±0.005	0.033±0.005	0.040±0.005	0.031±0.004	n.a.
*X. dorsalis* n = 4	0.137±0.01	-	0.016±0.003	0.016±0.003	0.012±0.003	0.0000
*X. brava* sp. nov. n = 3	0.145±0.01	0.134±0.01	-	0.017±0.004	0.00±0.002	0.0000
*X. atlantica* n = 6	0.127±0.01	0.142±0.01	0.095±0.009	-	0.012±0.003	0.0000
*X.* cf. *anselli* n = 3	0.136±0.01	0.126±0.01	0.041±0.006	0.083±0.008	-	0.0000
Within species (28SrDNA)	n.a.	0.002±0.001	0.000±0.000	0.0036±0.001	0.0098±0.003	-

p-values and standard deviation for 28SrDNA and 18rDNA are represented below and above the diagonal, respectively. n.a. =  not applicable, group with only one specimen.

For the 28S gene, *Xylophaga brava* sp. nov. displayed genetic distances of 9%,14.4% and 4% with *X. atlantica*, *X. dorsalis* and *X* cf. *anselli,* respectively. The intraspecific variation for 28S ranged between 0.00% in *X. brava* and 0.98% in *X* cf. *anselli* (Table 4).

The 18S gene was characterized by low interspecific divergences and close to zero intraspecific variations. Genetic distances between species were comparable among *X. dorsalis*, *X. brava* sp. nov. and *X. atlantica* (about 1.6–1.7%). The minimum genetic distance in 18S was found between *X. brava* sp. nov. and *X*. cf. *anselli* (0.5%). Although within-species, some nucleotides differed between individuals, intraspecific variability was always <0.001% (Table 4).

*Xylophaga brava* sp. nov. and *X*. cf. *anselli* seemed to be closely related but are distinct and separated in two different clades. The PTP method recovered six species of *Xylophaga,* with a moderate model fitting (Dtest: 1.182, P-value  = 0.000), and supported the hypothesis that *X. brava* sp. nov. and *X.* cf. *anselli* are two different species. This method however failed when applied to the 18S due to very poor model fitting (P-value>0.05) and all species, including the outgroups, were considered within a unique clade.

### Systematic Account

PHYLUM MOLLUSCA

CLASS BIVALVIA

SUBCLASS HETERODONTA

ORDER MYOIDA

FAMILY XYLOPHAGAIDAE [42]

GENUS *XYLOPHAGA* TURTON, 1822

#### Type species

*Teredo dorsalis* Turton, 1819 by original designation.

#### Diagnosis

*Teredo*-like shell lacking apophyses; with chondrophore and internal ligament. Body contained within the shell, siphons often able to retract between valves. Mesoplax divided, shape and size variable. Siphons entirely or partly fused, otherwise variable; excurrent siphon may be truncated. No calcareous tube [4].

### *Xylophaga dorsalis* (Turton, 1819)

*Teredo dorsalis* Turton, 1819.

*Xylophaga* A in Romano et al. [17].

#### Material examined

BC 900, 1200 and 1500 m depth: 1450 specimens; BC-OS 1200 m, 1500, 1800 m depth: 91 specimens; LFC 130 m depth: 845 specimens; LFC 1100 m: 473 specimens; AC 2000 m depth: 3 specimens; AC-WS 1200 m depth: 1 specimen. Additional material from the present study was deposited at MNCN (15.07/12214-12216) and at FMNH (342501-342507).

#### Additional Material Examined

BMNH: 4 lots of dry specimens only; PCMAG: 6 specimens, Eddystone and Plymouth Sound (UK) in 1908 and 1910 (2000.1.2921.1-4 and 3168.1-2); MOM: 25 specimens Azores, the Bay of Biscay and Tenerife in 1887-1888; LHD: 1 specimen, unknown locality; MBA: 2 specimens, Tamar estuary (UK) in 2009.

#### Extended Diagnosis

**Figure 4:**
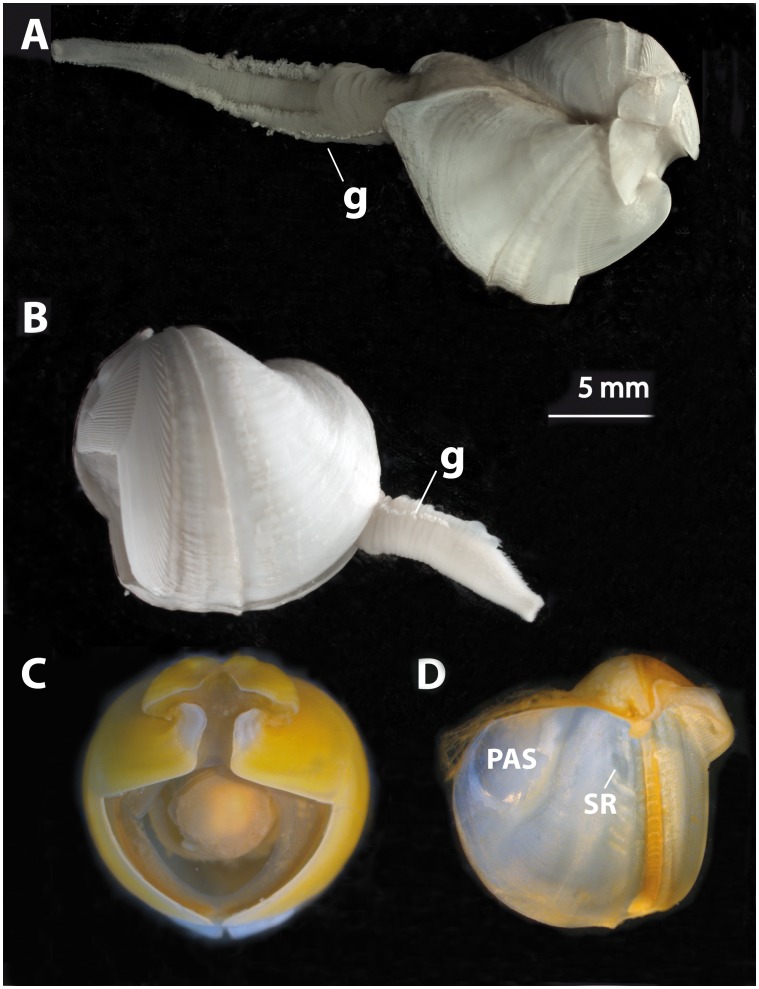
*Xylophaga dorsalis:* A) Dorsal view of shell with soft parts. B) Lateral view of shell with soft parts. C) Frontal view. D) Inner shell. g  =  white spots; PAS  =  posterior adductor scar; SR =  siphonal retractor scar.

**Figure 5:**
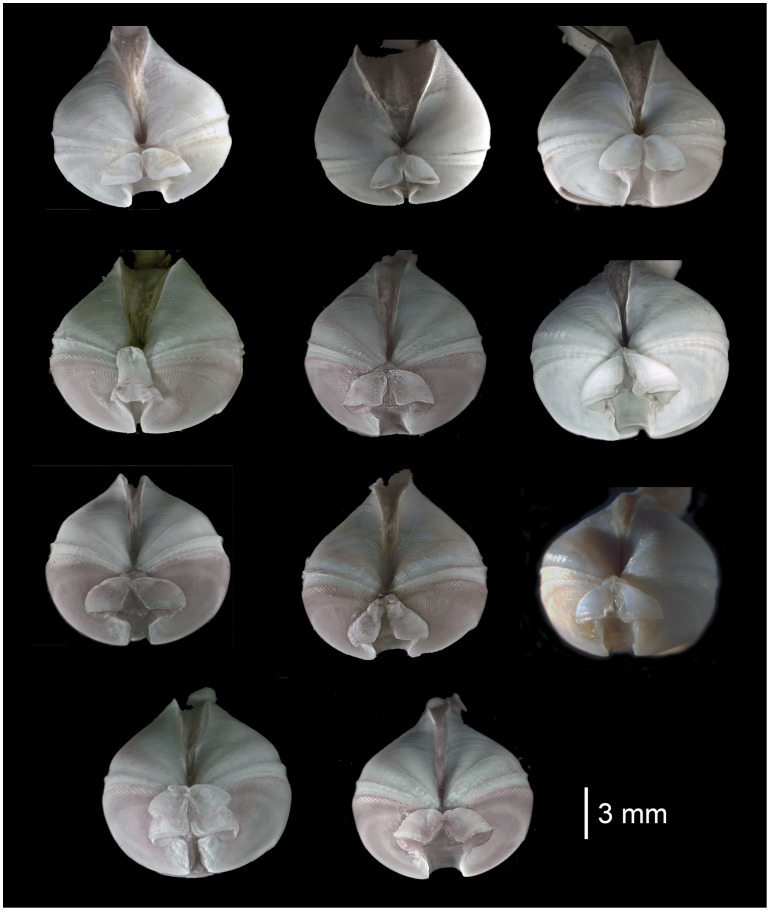
Variation of mesoplax among Mediterranean specimens of *Xylophaga dorsalis*.

Shell: rounded from 1.5 to 15.1 mm long, lacking posterior extension (Fig. 4); ear-shaped mesoplax highly variable in shape and size, with a sharp lateral edge (Fig. 5); beak non-projecting; umbonal-ventral sulcus with prominent posterior ridge, extending ventrally beyond shell margin. Inner shell: Shiny, poorly marked, oval posterior adductor scar (Fig. 4D). Faecal chimney present.

Excurrent siphon truncated near shell. Incurrent siphon with fringed lateral lobes on dorsal side and white spots set on line on lateral side (Fig. 4). Incurrent siphon 1.4 to 4.3 times excurrent siphon length (n = 60).

**Figure 6:**
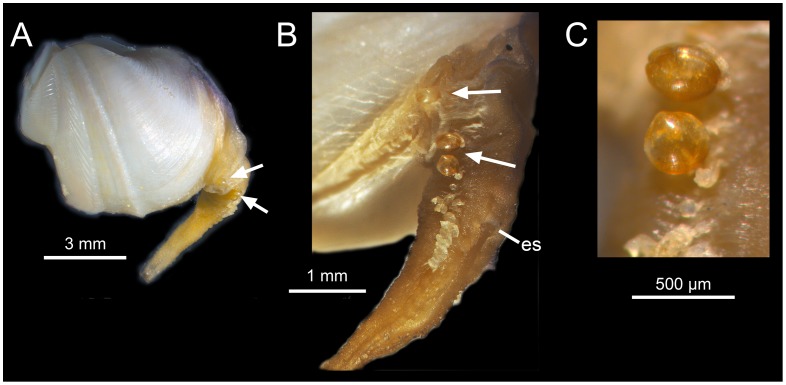
*Xylophaga cf. dorsalis* with small individuals attached to the siphons indicated by arrows. es  =  excurrent siphon opening In B, note the white spots on the lateral siphon.

Siphons: openings lack cirri, proper; typically with conspicuous dorsal fringed lappets on incurrent siphon distal to excurrent siphon opening; lateral and ventral parts of incurrent siphon usually with dense finger-like extensions of tissue, becoming more evident distally; white protruding spots at base of lateral siphon form a line extending distally beyond the excurrent siphon opening. These spots are hard in texture and they may fall off (Fig. 4 A, B). Two specimens from 1100 m depth at LFC with two-three tiny individuals (about 300 µm in diameter) attached to siphon near excurrent siphon opening (Fig. 6). These specimens were not included in the molecular analysis.

#### Remarks

A complete redescription of *X. dorsalis* is needed. We assigned these specimens to *X. dorsalis* based on their similarity to illustrations in Purchon [1] and Turner [43] and to specimens we examined from near the type locality. Turton's (1819) original description proved to be inadequate for a definitive identification. Turner [43] stated that the types of *X. dorsalis* were probably in the British Museum of London, but they are not. According to Warén (1993) Turton's collection was acquired by Jeffreys and later sold to the Smithsonian Institution (USNM); he recommended that any type material from Jeffrey's collection not present in the USNM should be considered to be lost. Despite this advice, we requested several museums check their holdings for potential types, to no avail.

The specimens we refer to *X. dorsalis* differ slightly from those illustrated in having a rounder, rather than a globular-oval shell, an umbonal-ventral sulcus with a prominent posterior ridge that extends ventrally beyond the margin of the shell and in lacking proper cirri at the siphonal openings (see below). Also, the white spots on the lateral siphon that were described as mucous glands forming a wedge-shaped mass by Purchon [1] are rigid and form in our specimens a line extending distally (Fig. 4 A, B).The mesoplax, once considered a conservative species-level character (Knudsen, 1961) in *Xylophaga* species, varies considerably in our specimens (Fig. 5), as had previously been reported among apparently conspecific Norwegian specimens [44], [45] and in *X. depalmai* Turner, 2002. The dense finger-like extensions of tissue on lateral and ventral incurrent siphon agree with those illustrated by Purchon (1941 Fig. 6b); these are likely transient structures similar to those in *X. multichela* Voight, 2008 illustrated by Reft & Voight (2009). In some views of some specimens, these can mimic cirri at the siphonal opening, but we do not consider them to form cirri proper.

Without geographical data, separating species that share an incomplete siphon, ear-shaped mesoplax, variable dorsal lappets on the incurrent siphon, a papillose incurrent siphon (features that we suggest are retractile and thus variable), and white spots on the lateral siphon (that can fall off) appears to be very difficult. Seemingly subtle characters of the mesoplax such as the sharp keel Turner [4] reported in *X. tipperi* or the triangle of hooks found by SEM in *X. multichela* may be important. Species that share these general characters in addition to *X. dorsalis* are those of Turner's (2002) Group 5: *X. depalmai, X. guineensis* Knudsen, 1961, *X. mexicana* Dall, 1908 (sensu Turner 2002), *X. tipperi* Turner, 2002, *X. bayeri* Turner, 2002, *X. globosa* Sowerby I, 1835, *X. japonica* Taki & Habe, 1950, *X. indica* Smith, 1904, and *X. multichela,* the latter having dwarf males attached to autonomously boring large ones, as do some of our specimens from LFC. The presence of dwarf males has been traditionally considered a species-specific character [46], further analyses are required to test this assumption.

#### Distribution

NW Mediterranean (BC, BC-OS, LFC), from 130 to 2000 m depth (Table 1), E Mediterranean at Nile Fan, 1690 m depth [20]; widely distributed in the Atlantic Ocean around the British Isles, from northern Norway (in the Norwegian Sea) to the Mediterranean [47], [48]. Literature reports of the species in the Western Atlantic from the Gulf of St. Lawrence to Cape Cod [47]–[49] are considered to be suspect, and may be better attributed to *X. depalmai* which is reported from as far north as Massachusetts, USA[4].

### *Xylophaga* cf. atlantica Richards, 1942

*Xylophaga* C in Romano et al. 2013.

#### Material examined

BC 900, 1200 and 1500 m depth: 20 specimens; BC-OS 1200, 1500 and 1800 m depth: 11 specimens; LFC 1100 m depth: 5 specimens; AC 2000 m depth: 3 specimens, AC-WS 1200: 3 specimens. Additional material from the present study was deposited at MNCN (15.07/12217 -12219) and at FMNH (342508-342510).

#### Extended Diagnosis

**Figure 7:**
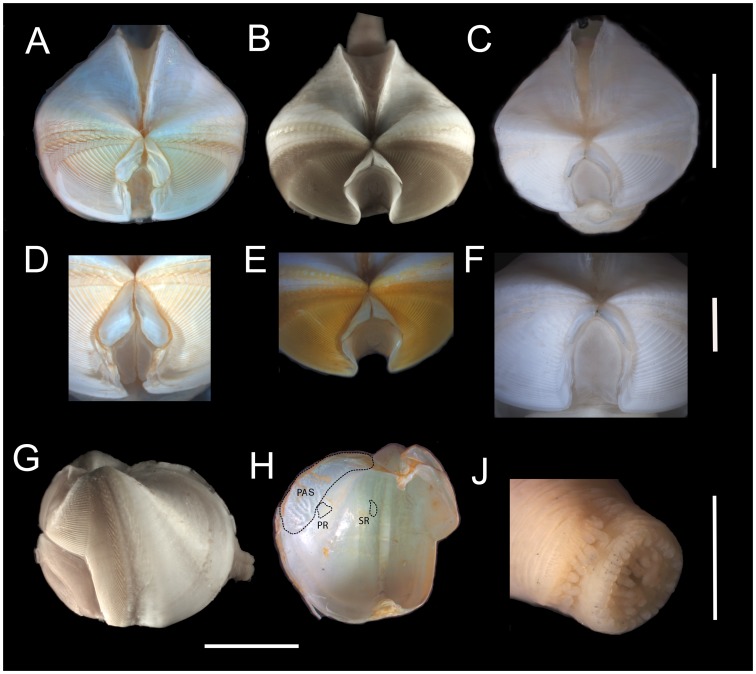
*Xylophaga* cf. *atlantica*. A-C: Dorsal views. D-F. Enlarged view of the mesoplax. G: Lateral view of shell with soft parts. H: internal shell. J: siphon openings. A,D,H: Morphotype C. B,E,G,J: morphotype D. C,F: morphotype E. Scale bar in A,B,C,G,H  =  5 mm; in D,E,F =  2 mm; in J  =  1 mm. PAS  =  posterior adductor scar; PR =  pedal retractor scar; SR =  siphonal retractor scar.

Shell ventrally bent toward inside at umbonal-ventral sulcus, subtly inflated posterior to sulcus. Shell with smooth ridge on either side of umbonal-ventral sulcus and length from 2.5 to 10.8 mm. Parallel bands of small spines on the concentric growth rings, visible on the posterior disk, may be present. Inner shell: posterior adductor scar with linear elements; pedal retractor scar evident (Fig. 7H); umbonal-ventral ridge mid-dorsally pronounced and thicker, narrows ventrally, with semi-circular condyle.

Mesoplax small with two plates set an acute angle to each other, ranging from trapezoid flat -vertically elongated plates (morphotype C Fig. 7A, D) to small triangular-projecting forward plates with long extensions forming a folded (morphotype D, Fig. 7B, E, G) or vertical support (morphotype E, Fig. 7C, F).

Siphons closely associated, generally shorter than shell length; excurrent siphon subterminal, opening dorsal to incurrent siphon, with a single outer band of 12-22 long and short cirri; incurrent opening with 2-3 rings of cirri. In morphotype D and E, incurrent siphon opens inside two rings of cirri: outer ring carries >20 stouter and big cirri, inner ring with 7–10 relatively long cirri (some cirri when viewed very closely appear to be bicuspidate) and a few smaller ones (Fig. 7J). In morphotype C, incurrent siphon opens inside three rings of cirri: outer ring with >20 very small, short papillae-like cirri, median ring with >10 small and thin cirri, inner ring with 6–8 relatively long, thin cirri.

#### Distribution

BC, LFC and AC from 900 to 1800 m depth (Table 4). Western Atlantic: Newfoundland, Canada south to Cape Henry, Virginia, USA from 15 to 1242 m depth (Turner, 2002). Species has also been reported from the Scotia Sea [50].

#### Remarks

Morphotypes C, D and E are each distinguishable based on the characters above However, the molecular results indicate that the one individual of type D sequenced cannot be distinguished from Morphotype C and they group together with *X. atlantica* from GenBank. Unfortunately, we had no available material of morphotype E to carry out molecular analyses. All these morphotypes share the mesoplax characters and subequal siphons with cirri at both openings with species of Turner's Group 4 [4]. The variation here is perplexing, but overall the specimens are consistent with *X. atlantica* Richards, 1942. Morphological variation has not been discussed within the xylophagaids, however, Turner [4] admitted to having confused *X. atlantica* with her *X. clenchi* Turner, 2002 in discussing the occurrence of what she termed brooded young, now hypothesized to be dwarf males. She may also have done so in reporting the number of papillae at the excurrent opening in 1955 [43] which differed from the value she reported in 2002. No dwarf males were found on individuals of morphotypes C, D or E.

Designating these specimens as members of *X. atlantica* is conservative as it is in accordance with GenBank; it is the senior name, if *X. atlantica* and *X. clenchi* are eventually found to be synonyms. Given that the holotype of *X. atlantica* is a dry shell (Richards, 1942) and [51] reported that the best way to distinguish the species was by the siphons, the matter will not be readily resolved.

Analyses including other genes and additional specimens of each morphotype are needed to confirm that they are conspecific. Among the other species of Turner's (2002) Group 4, *Xylophaga abyssorum* Dall, 1886 is unique in having a prominent ridge posterior to the umbonal-ventral sulcus. *Xylophaga muraokai* Turner, 2002 has an excurrent siphon opening with cirri differing in size from mid-dorsal to ventral, and an incurrent siphonal opening with cirri of three types. In our specimens, the excurrent cirri are mixed (long and short) and the incurrent ones are of two types. *Xylophaga foliata* Knudsen, 1961 and *X. duplicata* Knudsen, 1961 are characterized by having a mesoplax with longitudinal folds and being erect (standing off the surface of the valves), respectively. *X. duplicata* also has a smooth posterior adductor scar, and only 6-8 cirri at each siphon opening. Both species can be easily distinguished from our specimens, which have a smaller mesoplax, a marked posterior adductor scar, and many more cirri. The siphons of *X. grevei* Knudsen, 1961 are similar to those of our specimens, but that species has an elongated shell and incurrent and excurrent siphon openings with 35 similarly-sized cirri and only 6 cirri, respectively.

### *Xylophaga brava* sp. nov. Romano, Pérez-Portela and Martin

**urn:lsid:zoobank.org:act:742686D2-B4AD-431F-BA73-1A26713CBE07**

*Xylophaga* B in Romano et al. (2013).

#### Material examined

BC 900 m, 1200 m, 1500 m depth: 261 specimens; BC-OS 1500 m, 1800 m depth, 12-month: 23 specimens; LFC 1100 m depth: 47 specimens.

#### Type material

*Holotype*: Museo Nacional de Ciencias Naturales de Madrid (MNCN 15.07/15003); Blanes Canyon, Spain 41° 27' 29'' N 2° 52' 58'' E, 1500 m depth in pine 12-month deployments. Measurements of holotype: Shell length 7.1 mm, height 7.0 mm, width 6.9 mm, siphons length 12.1 mm, proximal part length 6.5 mm. *Paratypes*: 4 specimens(FMNH 328599-328600) and 7 specimens (MNCN 15.07/15004-15005), Blanes Canyon, 900–1500 m in pine deployments. Other voucher material was deposited at Centre d'Estudis Avancats de Blanes (CEAB).

#### Etymology

Species name referring to “Costa Brava” ( =  “wild coast” in Catalan), the NW Mediterranean Iberian coast along which the specimens were first found.

#### Diagnosis

Shell generally dorso-ventrally elongated, with pronounced constriction posterior to umbos. Mesoplax weak, poorly mineralized, only sometimes erect. Siphons up to three times shell length; united throughout. Proximal and distal siphons distinct: proximal weakly muscled, with translucent cover; distal muscular region with concentric rings. Both siphons open within a ring of external cirri, inside this, the excurrent siphon opens within a ring of cirri, the incurrent often with a small protruding tube.

#### Description

**Figure 8:**
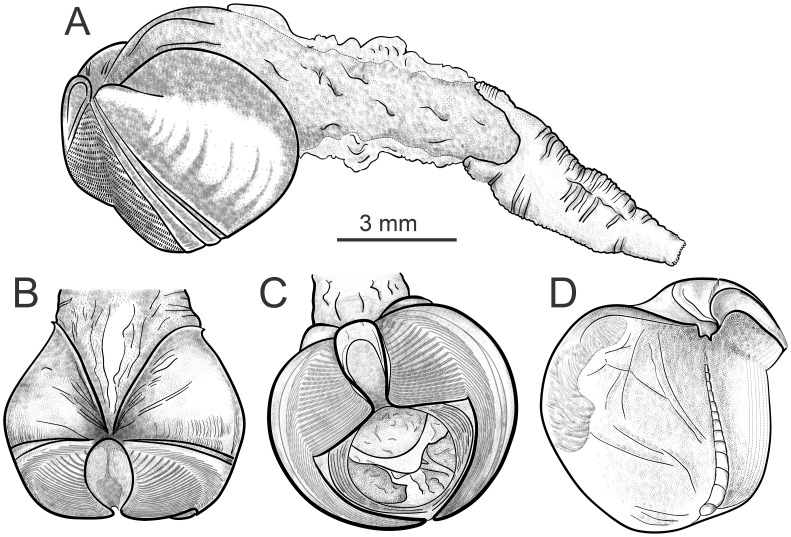
*X. brava:* n. sp. A) Lateral view of shell with soft parts. B) Dorsal view. C) Frontal view. D) Inner shell where shadow area represents the posterior adductor scar.

Shell: thin, from 1.5 to 9.3 mm long and high (Fig. 8A). In dorsal view, shell laterally constricted posterior to umbos (Fig 8B). Ventral shell depressed where condyles meet. Umbonal-ventral sulcus poorly developed. Umbos taller than posterior shell; when erect, mesoplax can be tallest feature. Inner shell: umbonal-ventral ridge emerges gradually to form condyle at ventral shell margin (Fig. 8D). Posterior adductor scar poorly marked, best seen in intact specimens; scar shiny in dry shells, restricted to dorsal of shell, appearing as series of short lines oriented at 45° to dorso-ventral shell axis. Pronounced but short ridge in internal dorsal shell between umbonal-ventral ridge and posterior adductor scar.

Mesoplax: poorly calcified, if at all; two parts usually not readily distinguishable; considerable amount of associated membranes; best observed after removing the membrane; forms a tall, open U-shaped extension with associated membranes when erect. Umbonal reflection comparatively small, simple margin around beak opening to which it closely adheres, most extensive dorsally, lateral to umbo. Small prodissoconch II, barely visible at the stereomicroscope, 180 ± 20 µm long (n = 25).

Anterior adductors contracted in nearly all specimens, sometimes strongly so, resulting in gaping posterior valves. Caecum readily visible between valves, linear in most specimens, maybe loosely coiled.

**Figure 9:**
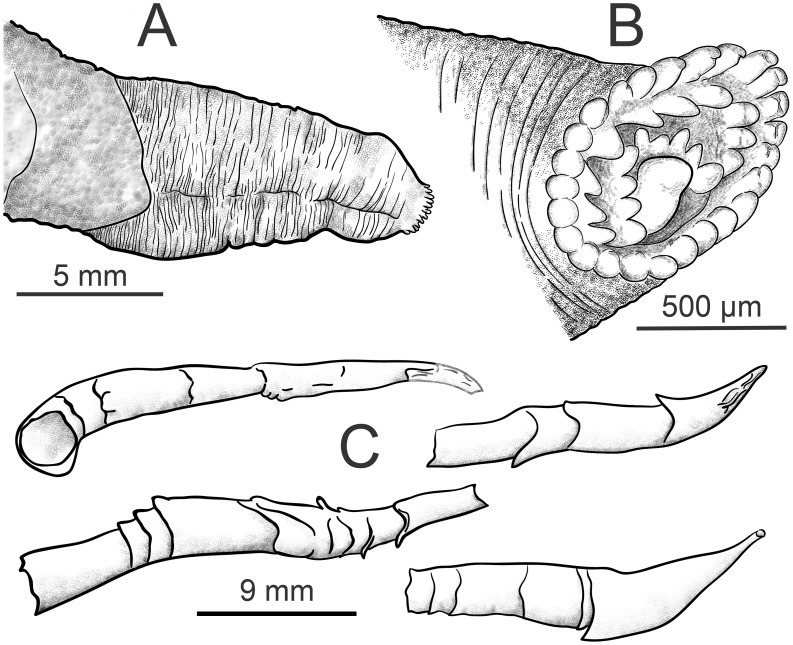
*X. brava* n. sp*:* A.)Enlarged view of the distal siphons. B) Common siphon opening. C) cones which cover the siphons.

**Figure 10:**
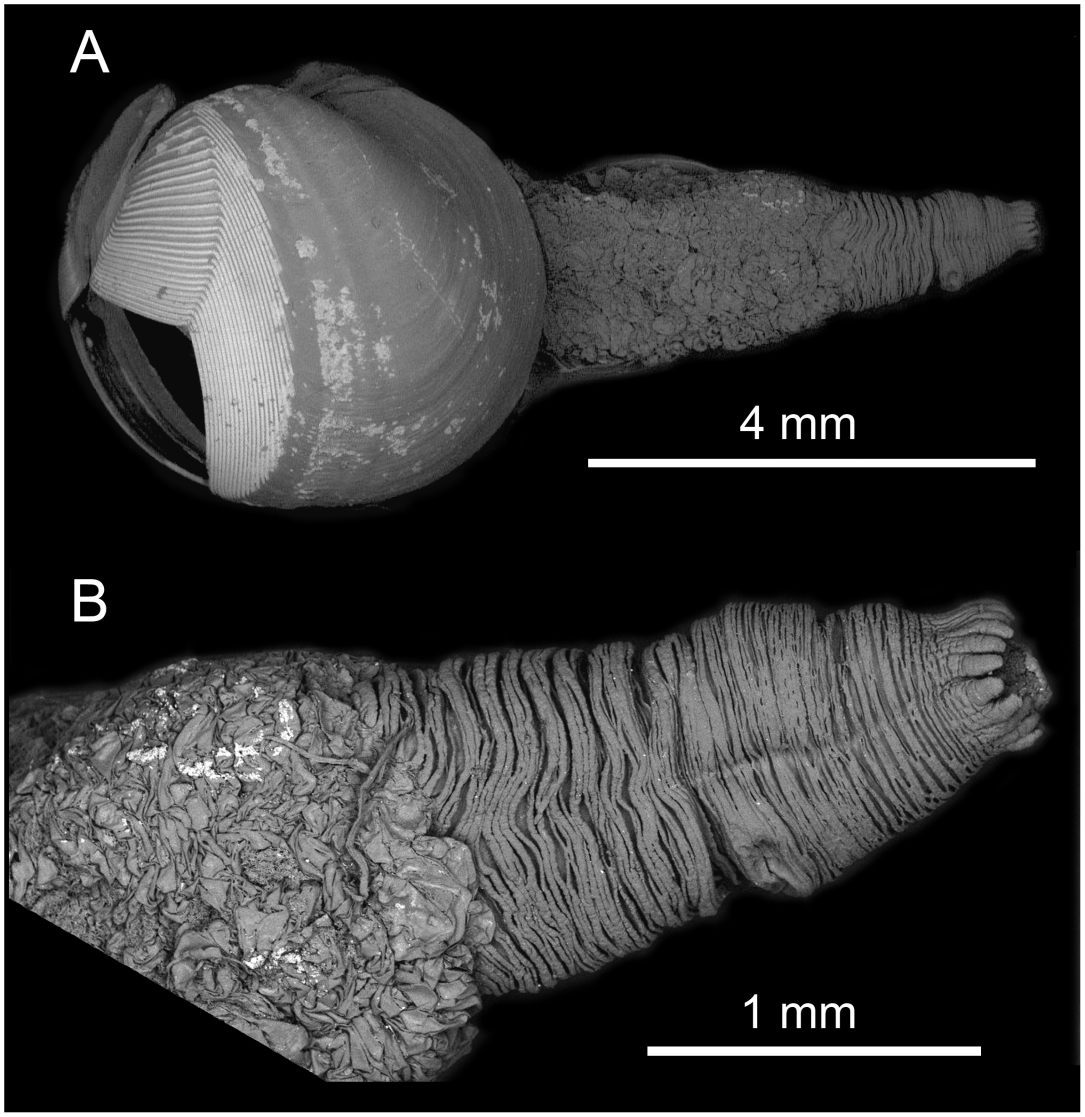
*X. brava* n. sp*:* A) SEM micrograph of the whole animal from left side. B) enlargement of the distal siphons.

Siphon: 1.5 to 3 times shell length; united throughout. Proximal siphon weakly muscled (Fig. 8), translucent in some specimens, covered with transparent light gold membrane, generally longer than the distal; distal siphon densely muscled, contracted into concentric rings (Fig. 9 and Fig. 10). The two parts meet in a clearly scalloped margin (Fig. 8A and 9A). Both siphons open inside one ring of 16–22 large cirri; internally one siphon opens within a ring of 18–20 cirri and the other (in some specimens) with a small protruding tube (Fig 9B). Periostracal cone over siphons often collected separately (Fig. 9C). Distal cone often smooth, proximal cone thicker and consists of multiple layers. The whole cone appears uniformly smooth and transparent after careful removing multiple layers. Faecal chimney present.

#### Distribution

NW Mediterranean at BC, BC-OS, and LFC, from 900 to 1800 m depth (Table 1).

#### Remarks

The two-parted siphon may be somewhat suggestive of species in the genus *Xyloredo* Turner, 1972, recently redescribed by Haga & Kase [52], which also have a strongly reduced mesoplax. However, our specimens do not have a calcareous tube lining their burrows. This, together with all remaining morphological characters, defines our species as belonging to *Xylophaga*.

*Xylophaga brava* sp. nov. resembles *Xylophaga heterosiphon* Voight, 2007 in having distinctly two-parted siphons, poorly calcified mesoplax, siphonal openings within a common ring of cirri, thin shell, and periostracal cones. The two species differ in: 1) the muscle texture of the proximal siphon is notably weaker, even transparent in *X. brava* sp. nov. and comparatively denser in *X. heterosiphon*; 2) the posterior adductor muscle scar is clearer in *X. heterosiphon* than in the new species; 3) a ridge in the inner shell extends from near the umbo to the posterior-ventral margin in *X. heterosiphon,* in *X. brava* sp. nov. the strongly-defined ridge is short and 4) the anterior umbonal reflection in *X. heterosiphon* is much reduced.

*X. brava* sp. nov. resembles *X. gerda* Turner, 2002 from 284 m depth off Grand Bahama Island, in having the shell with a concave posterior slope, tiny mesoplax, equal length siphons with a periostracal sheath, generally smooth and glisteny inner shell, a weakly marked posterior adductor scar, and a low umbonal-ventral ridge. However, *X. brava* sp. nov. can be distinguished from *X. gerda* by its distinctly different proximal and distal siphons, its mesoplax that can extend beyond the umbos when erect, its siphons that are united through their length and that both siphonal apertures open within a single ring of cirri.

*X. brava* sp. nov resembles *X. concava* Knudsen, 1961: notably shell shape, erectable mesoplax, and long siphons that open close to each other. However, *X. concava* differs in having apparently a periostracal cover over the entire siphon, as opposed to a cone, an essentially semicircular mesoplax, and attached juveniles [53]. X*ylophaga brava* sp. nov. also resembles *X. anselli* Harvey, 1996, a species described from Atlantic Scottish waters based on a few, very small individuals (<1 mm). However, the former differs in having often weakly muscled and transparent proximal siphon, a smaller prodissoconch II (see the description of *X.* cf. *anselli*), more cirri at the siphon openings, and a rounder posterior shell margin. The specimens of morphotype F from AC in the Cantabrian Sea are geographically and morphologically closer to *X. anselli* (see the description of *X.* cf. *anselli*). Moreover, our molecular data show the respective specimens of both species grouped in two different, well-supported clusters, thus confirming the genetic isolation between them.

### *Xylophaga* cf. anselli

#### Material examined

AC-WS 1200 m depth: 2 specimens; AC 2000 m depth: 16 specimens. FMNH312286.

#### Diagnosis

**Figure 11:**
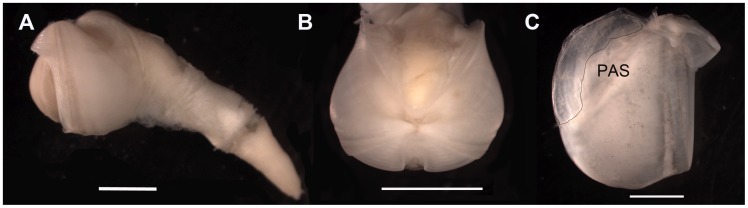
*X.* cf. *anselli:* A) Lateral view of the whole animal. B) Dorsal view. C) Internal shell. PAS  =  posterior adductor scar. Scale bar in A  = 2 mm, in B and C  = 3 mm.

Shell length in our specimens from 1.8 to 8.5 mm. Shell thin, anterior beak narrow. Umbonal-ventral sulcus only slightly depressed. Posterior shell margin vertical, dorsally and ventrally equally developed. On the inner shell, umbonal-ventral ridge emerges gradually at ventral shell margin (Fig. 11). Posterior adductor scar large (Fig. 11C).

Siphons: 1 to 1.7 times shell length; united throughout. Proximal and distal siphons distinct: proximal opaque and wrinkled; distal densely muscled, contracted into concentric rings. The two parts meet in a linear margin. Both siphons open in one ring of up to 15 cirri. Periostracal cone that covers at least the distal siphons may be present.

**Figure 12:**
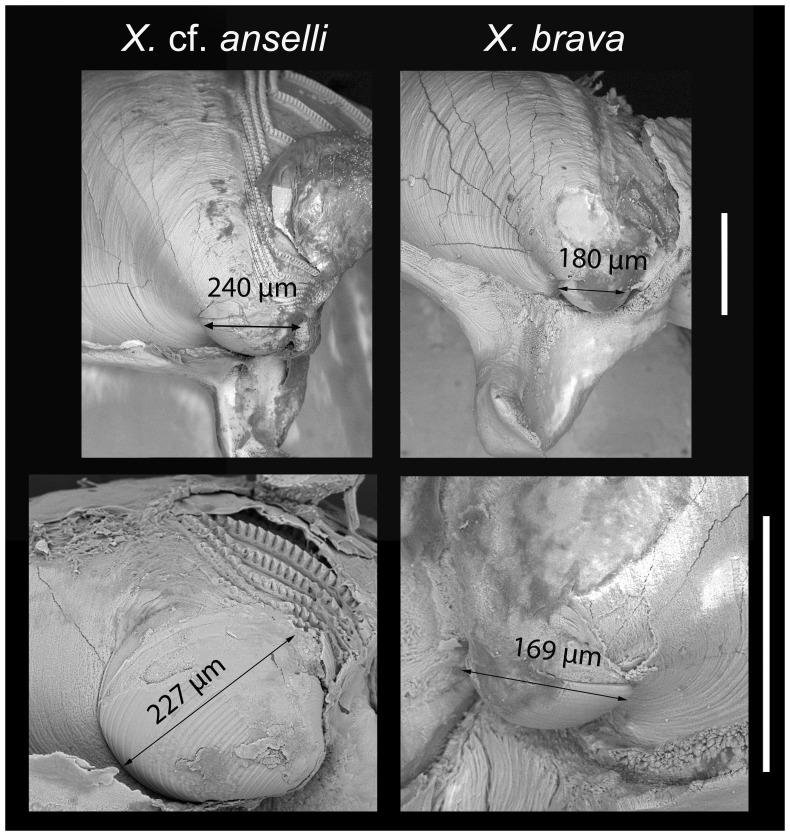
SEM micrographs of umbonal areas of specimens of *X.* cf. *anselli* (left) and *X. brava* (right) Arrows measure prodissoconch diameter. Scale bars  = 250 µm

Mesoplax: poorly calcified, if at all; two plates not seen. Amber brown prodissoconch II always visible at the stereomicroscope, 240 ± 15 µm long (n = 6) (Fig. 12) in agreement with Harvey (1996) and with observation of the FMNH specimen that had a visible prodissoconch, 200 µm long.

#### Remarks

We assigned these specimens to *X.* cf. *anselli* based on their morphological similarity to the original description by Harvey (1996) and comparison to FMNH 312286, which was collected at the type locality.. However, all Harvey's specimens were less than 1 mm in shell length, making it impossible to compare the adult characters to those in the juveniles. The larger specimen in FMNH 312286 shows a trace of a periostracal cover on the proximal siphon. Harvey [54] did not distinguish two separate siphon parts, perhaps because of their size, the separation was not apparent; he described only one siphon opening and did not discuss whether both siphons could open in a single ring of cirri. He also reported up to 12 cirri in the siphon opening, while our specimens show up to 15 cirri. However, we suggest that cirri number may increase with increasing size. A complete redescription of *X. anselli* based on specimens of a wide series from 1 mm to full adult from the type locality is thus required.

*Xylophaga* cf. *anselli* from the Cantabrian Sea, Atlantic Ocean differed from *X. brava* sp. nov., Mediterranean Sea, in having a proximal siphon which is denser and more compact, never transparent; fewer cirri at the external siphon opening (up to 15 vs. 22 in *X. brava* sp. nov. in adults of similar size) and a larger prodissoconch (240 ± 15 µm *vs* 180 ± 20 µm in *X. brava* sp. nov., Fig. 12).

*X. anselli* differs from *X. heterosiphon* in frontal view by the larger umbonal reflection that extends more ventrally in the former species than in the latter. A limited number of poorly preserved specimens in the collections of the United States National Museum (USNM 757359; 757342) indicate that this or an overtly similar species occurs in the western Atlantic Ocean at depths of 2915-3090 m off Norfolk, VA USA.

## Discussion

Few studies investigated the evolutionary relationship among members of Pholadoidea [40], [55], [56] and fewer still have tried to integrate morphological and molecular studies in the taxonomy of marine wood borers of the Teredinidaes [57], [58]. However, as far as we are aware, molecular tools have never been used in taxonomic studies to assist in species delimitation among xylophagaids.

Our combined morphological/molecular study demonstrated the existence of several species of *Xylophaga* in Iberian Peninsula canyons and increased significantly our knowledge about the distribution and morphological plasticity of some of the species found. Our study also recorded various degrees of separation among the Atlantic and the Mediterranean forms of *Xylophaga* spp.

### Systematics

Regarding *Xylophaga. dorsalis*, we observed subtle morphological differences between our morphotype A and previous descriptions of the species based on specimens from the SW British coasts. Comparison of molecular data with voucher specimens was not possible because attempts to sequence material from museums were unsuccessful due to degraded DNA and the poor quality of the extractions. However, we considered these differences insufficient to assign species-level status. Accordingly, the respective AC (Atlantic) and BC and LFC (Mediterranean) specimens were found to be genetically inseparable. Considering that the type material is missing, there remains the concern that a complete redescription of *X. dorsalis* is required.

Similar results were found for *X. atlantica*. Although slight morphological variation occurred among our specimens (morphotypes C-E), the respective sequences of morphotype C and D grouped all together and formed a single clade with *X. atlantica* from the West Atlantic. No clustering of sequences from different canyons was seen (Fig. 3) and intraspecific variation was low (Table 4). Unfortunately we had no sequences of morphotype E for comparison but we have no evidences to demonstrate they belong to a different species from the other morphotypes of *X. atlantica.*

In contrast, *X. brava* sp. nov. and *X.* cf. *anselli*, arose as two sibling species with few morphological differences, inhabiting respectively Mediterranean and Atlantic submarine canyons and clearly representing different taxonomic units. These two species are morphologically similar, deriving from a recent ancestor, but genetically isolated in two different and vicariant evolutionary units. Additionally, interspecific divergences for both genes, the 28S and 18S, were significantly higher than the intraspecific variability observed. Our results confirm that the 18S and 28S genes are highly conserved (the former more than the latter), making our conclusions about *X. brava* sp. nov. and *X*. cf *anselli* more robust. The variability levels among *Xylophaga* spp. are comparable to those reported for other bivalves, which also lack 18S intraspecific variation [59], and support the hypothesis that small distances (around 0.3%) among allopatric specimens are evolutionary meaningful [57].

Therefore, the divergence between *X*. *brava* sp. nov. and *X.* cf. *anselli* and their grouping in two well-supported clades show the absence of interbreeding between the Mediterranean and the Atlantic evolutionary lineages, suggesting that this situation has been stable over time leading them to split into different species. The Atlanto-Mediterranean littoral region experienced an intricate geological and climatological history [60]–[62]. The Gibraltar Strait, the only connection between the Atlantic and the Mediterranean, has been one of the most important potential biogeographical breakpoints in the world's oceans due to its strong current regimes [63], [64], and repeated disconnections and reconnections over geological timescales. As a consequence a number of speciation processes and genetic divergence between the basins was promoted [64]–[67]. Hence nowadays the Cantabrian and Mediterranean Seas, where *X.* cf. *anselli* and *X*. *brava* sp. nov. were found, belong to different biogeographical Ecoregions, the Lusitanian South-European and Mediterranean regions [68].

From a morphological point of view, we found some differences between *X*. *brava* sp. nov. and *X.* cf. *anselli,* but to some extent, differences in some life strategy may have contributed to promote evolutionary differentiation of the two species. For instance, the smaller prodissoconch in *X. brava* sp. nov. suggests the existence of larvae with a shorter planktonic stage and, consequently, reduced dispersal potential. Moreover, speciation is not always accompanied by large morphological divergence [69]–[71], particularly in recent episodes or under very extreme environmental forcing [22], which exert similar pressures to the isolated populations.

The original description of *Xylophaga anselli* was based on small specimens (juveniles) from the North-East Atlantic coast of Scotland [54]. Lacking adequate material of this species to allow a complete description based on adult specimens from the type locality, as well as molecular analyses, we decided to report the Cantabrian specimens as *X.* cf. *anselli* in accordance to their morphological similarity and geographical proximity, while we describe the isolated Mediterranean species as *X. brava* sp. nov.

The combined results of our morphological and molecular data also suggested that further studies including more species of *Xylophaga* (borehole lining absent, well-developed, calcified mesoplax) and *Xyloredo* (calcified borehole lining, reduced mesoplax), should be addressed to resolve the relationship among these two genera, particularly taking into account that species such as *X anselli*, *X. heterosiphon* and *X. brava* sp. nov., showed intermediate morphological characters (i.e. organic borehole lining, small uncalcified mesoplax) and turned to be clustered within *Xyloredo* and *Xylopholas* in our molecular analyses.

### Diversity of *Xylophaga* species in Iberian canyons

The presence of three species of *Xylophaga* in two NW Mediterranean deep-sea submarine canyons increases the recognized diversity of the genus in the Mediterranean by a 200%. Only *X. dorsalis* had been previously reported from that sea, initially from the Iberian coasts (Jeffrey, 1865) and, more recently, from the Nile Fan (Eastern Mediterranean) at 1640 m depth [19], [20]. Among the species with Atlantic distribution, *X. anselli* was heretofore known only from the Northeast Atlantic type localities (Hebridean slope and Anton Dohrn Seamount, 1370–2195 m depth) [54]. Thus, our discovery of the species in the Cantabric Sea would extend its geographical distribution to the south.

*Xylophaga dorsalis* was by far the most abundant species, based both on the colonized deployments (nearly all) and the number of individuals in each deployment, this abundance being consistent with the previous reports from Mediterranean locations [17]. *Xylophaga brava* sp. nov., the second species in abundance, was much more common in pine than in oak and the available data suggested it is restricted to the Western Mediterranean, more studies are needed to better define its distribution. Specimens attributed to *X. atlantica* were comparatively rare, but they occurred in all canyons (Fig. 1). The species had previously been reported only from the West Atlantic [4]. The shallow-water wood-borer, *Teredothyra dominicensis*, previously considered to be a Caribbean endemism, has also been reported from the Mediterranean [57]. Whether the presence of these species indicates recent invasions or simply reflects an increasing number of available samples or of the taxonomic expertise, both leading to a better knowledge of the species distribution, cannot be currently assessed.

Our results confirm that deep-sea environments still host a large amount of undiscovered fauna. Particularly, submarine canyons, where sedimentation and accumulation of organic debris are enhanced, may represent hot spot of biomass and endemism [17], [72], [73] and therefore should be recognized as potential high priority areas for the conservation of the deep sea. The 4700 m depth site from AC appears to be exceptional in that none of the wood cubes deployed there were colonized by *Xylopaga*, or any other macroinvertebrate. This may be due to unfavorable local circulation patterns, extreme environmental factors, or the lack wood as potential food source to maintain a stable population of wood borers.

Our results refute the long-held view that larvae of *Xylophaga* do not colonize wood that is not in contact with, or in close proximity to, the seafloor [74], only Turner [4] previously reported that few specimens of *X. depalmai* were recovered up to 90 m from the bottom but she considered the event very unusual. *Xylophaga* species seem to disperse higher in the water column than do other deep-sea benthic species associated with ephemeral, fragmented habitats such as organic falls. Specimens of the bone-eating *Osedax* worms, for example, decreased drastically one meter above the sediment floor [75]. Moreover specimens of *Idas* Jeffreys, 1876, a deep-sea bivalve generally associated with wood falls, were found in wood deployments at the sediment surface but were never recovered in suspended ones at the same site [17].The ability of larval *Xylophaga* to disperse higher in the water column, and therefore to be subject by transport of stronger currents, may be a key factor in allowing the persistence of the adult populations despite living in patchy wood fall habitats.

Future studies focusing on ecology and populations' connectivity inside and outside canyons, as well as between other deep-sea environments, are required to assess the real larval dispersal and colonizing abilities of these highly specialized organisms. To assess populations' connectivity among hot spots of biomass and endemisms such as submarine canyons will be fundamental for the development of a sustainable management strategy of the deep-sea ecosystems and its associated resources [76] and will certainly aid in the scientific design of marine protected areas.
